# Sex Differences in Clinical Characteristics and Brain MRI Change in Patients With Wilson’s Disease in a Chinese Population

**DOI:** 10.3389/fphys.2018.01429

**Published:** 2018-10-09

**Authors:** Xiaohu Li, Zhiqiang Feng, Wei Tang, Xuen Yu, Yinfeng Qian, Bin Liu, Xiaoshu Li, Renmin Yang, Yongqiang Yu

**Affiliations:** ^1^Department of Radiology, The First Affiliated Hospital of Anhui Medical University, Hefei, China; ^2^Department of Neurology, The Hospital Affiliated of Anhui College of TCM, Hefei, China

**Keywords:** Wilson’s disease, MRI, gender, Chinese patients, clinical characteristics

## Abstract

**Background:** Wilson’s disease (WD) is an inborn copper metabolism disease. Sex differences in clinical features of WD patients have been reported; however, the effect of sex on brain MRI is still unclear, especially for Chinese WD patients. Therefore, we aimed to examine sex differences in clinical correlates and brain MRI changes in WD patients in a Chinese Han population.

**Methods:** 535 WD patients were enrolled and underwent MRI scanning. These patients were subdivided by the clinical symptoms, Kayser–Fleischer (K–F) rings, laboratory tests and sex. The mean age of onset and diagnosis, disease latency, localization of brain MRI lesions, and the level of copper metabolism were compared between male and female patients.

**Results:** The neuropsychiatric form (452 and 84.5%) was the most common subtype. Compared to female patients, male patients had a higher percentage in three clinical forms: neuropsychiatric form (263 and 58.2%), hepatic form (41 and 59.4%), and presymptomatic form (10 and 71.4%). In the neuropsychiatric form, male patients had the earlier age of onset and definitive diagnosis, and shorter time of disease latency than female patients. Putamen was the most common site for lesions in brain MRI of three groups. In the hepatic form, more male patients showed the ventricular widening than female patients (14/41 vs. 3/28; *p* < 0.05). The level of serum ceruloplasmin and copper of WD patients with neuropsychiatric form was higher than that of male patients with hepatic or presymptomatic form. In women, however, patients of presymptomatic form have the highest level of the ceruloplasmin, and the level of serum copper in hepatic patients was highest.

**Conclusion:** Our findings suggest sex differences in the percentage of three clinical forms. Meanwhile, the mean age of onset and diagnosis of female was higher than male, also happened in the disease latency. Only in the hepatic form, there was a sex difference in the ventricular widening.

## Introduction

Wilson’s disease (WD), also called as hepatolenticularis, a rare autosomal recessive inherited disease with a prevalence rate of about 1 in 30000 people (El-Youssef, 2003; Huster, 2010; Bandmann et al., 2015). The correlation between WD and copper transport was demonstrated in 1948 (Mandelbrote et al., 1948) and the pathogenic gene *ATP7B* was confirmed and cloned in 1993 (Petrukhin et al., 1993). Copper metabolism disturbance as a result of null mutation of the *ATP7B* gene located on chromosome 13 influences the production of ceruloplasmin and then leads to an excessive accumulation of copper in the liver, kidneys, brain and other organs (Fatemi and Sarkar, 2002; Jayakanthan et al., 2017). Furthermore, the abnormal accumulation of copper causes a series of clinical manifestations, such as jaundice, dysarthria, clumsiness, tremor, drooling, gait disturbance, malaise, and arthralgia (Stremmel et al., 1991; Ferenci, 2004; Machado et al., 2006).

Magnetic resonance imaging (MRI) is a non-invasive and sensitive detection tool for WD patients. Previous MRI findings in WD revealed ventricular widening, cerebellum and cortex atrophy and lesions in the putamen, globus pallidus, caudate nucleus, thalamus, mesocerebrum, pons, medulla, and cerebellum. The brain MRI showed decreased signal intensity on T2-weighted imaging, which could be explained by the paramagnetic effect of copper accumulation (Mironov, 1991; van Wassenaer-van Hall et al., 1996). For diffusion weighted images (DWI), WD patients exhibited decreased diffusion in the putamen before the neurologic symptoms were present, while they exhibited increased diffusion after neurologic symptoms (Favrole et al., 2006). By far, only a few studies performed the brain MRI tests in WD patients and examined the correlation between MRI lesions and symptoms. However, the sample size of those studies was relatively small, with mixed results (Magalhaes et al., 1994; King et al., 1996; Saatci et al., 1997).

Sex differences in brain MRI in healthy people have already been reported. For example, males have heavier and larger brains than females. Males have larger volume mainly in frontomedial cortex, hypothalamus, and the amygdala, while females have lager volume mainly in frontal and medial paralimbic cortices. Moreover, the percentage of gray matter in the whole brain is higher in men than in women; however, women have higher proportions of white matter than men. It was reported (Zaidi, 2010) that the sex hormones (androgen and oestrogen) played a crucial role in structural differences between men and women.

Recently, evidences showed sex differences in neurodegenerative diseases (Nasrallah et al., 1990). Litwin found that female tended to present with globus pallidus lesions more often than male, on the contrary, more male than female showed cerebellar atrophy in neuropsychiatric form and cortical brain atrophy in presymptomatic form (Litwin et al., 2013). However, no study has reported sex differences in clinical phenotypes and MRI data in a large sample size of Chinese WD patients, which was examined in this study.

## Materials and Methods

### Methods and Subjects

The study was approved by the local ethics committee. 535 patients (314 men and 221 women) who were newly diagnosed as WD were recruited in this retrospective study from 2006 to 2013.

There were 452 patients (263 men and 189 women) for neuropsychiatric form, 69 patients (41 men and 28 women) for hepatic form and 14 patients (10 men and 4 women) for presymptomatic form. The WD diagnosis was based on the clinical symptoms, Kayser–Fleischer (K–F) ring, the levels of serum ceruloplasmin and copper, 24 h urine copper excretion and MRI examination. All the patients were not treated before the diagnosis. The levels of copper metabolism were measured, and then remeasured by different groups of technicians who were blinded to any information of the subjects.

In the meanwhile, a conventional brain MRI test was carried out in all patients. All the images, including T1- and T2-weighted and FLARE sequences, were analyzed by two experienced radiologists who were blind to any other clinical information. Lesions, encephal atrophy, ventricular widening, and encephalomalacia were found in all patients. The lesions were detected in putamen, globus pallidus, caudate nucleus, thalamus, mesocerebrum, pons, medulla, and cerebellum. The encephal atrophy was segmented into two groups: cerebellum atrophy and brain cortex atrophy.

Generally, the typical clinical symptoms, laboratory test (lower serum ceruloplasmin, higher serum copper, and urine copper excretion), and gene mutation were needed for the diagnosis of WD. The patients were divided into three subtypes: (1) neuropsychiatric form: the combination of K–F rings, laboratory tests, and extrapyramidal symptoms were used to confirm this subtype directly; (2) hepatic form: the combination of K–F rings and laboratory tests were used to confirm this subtype directly; and (3) presymptomatic form: gene mutation was confirmed by molecular genetic examination (Ferenci et al., 2003). The predominance symptoms scoring system at diagnosis was the same as previous study (Merle et al., 2007). Neuropsychiatric symptoms include: tremor, salivation, dysphagia, speech, writing and gait disturbances, adynamia, epileptic seizures, involuntary movements, mood disturbances, cognitive impairment, were evaluated and ranged from 0 (completely normal) to 3 (severely impaired). Hepatic symptoms include: Weakness, anorexia, lower extremity edema, jaundice, hematemesis, abdominal distension, hemorrhages, and fulminant liver failure, were also evaluated and classified into four categories: 0 (completely normal); 1 (increased levels of liver enzymes, without signs of liver cirrhosis or impaired liver function); 2 (compensated liver cirrhosis); and 3 (decompensated liver cirrhosis or acute liver failure). The type of patients with a hepatic or neurological score ≥1 hinge on the higher score, patients with a neurological and hepatic score of 0 were grouped into presymptomatic form, and patients with the same score (>0) were grouped into neuropsychiatric form (Merle et al., 2007).

Firstly, we analyzed the distribution of provinces where these Chinese WD patients were from. Secondly, male/female ratio, age of onset, and diagnosis, disease latency of three clinical types were examined. Then, the sex differences in brain MRI data in each group were compared. The brain MRI changes were divided into twelve groups: putamen lesions, globus pallidus lesions, caudate nucleus lesions, thalamus lesions, mesocerebrum lesions, pons lesions, medulla lesions, cerebellum lesions, cerebellum atrophy, ventricular widening, brain cortex atrophy, and encephalomalacia. Finally, the serum levels of copper and ceruloplasmin were analyzed.

The same group of neurologists and radiologists in our hospital carried out the clinical classifications.

### Statistical Analysis

The mean values, range, percentage, and standard deviation (SD) were described. Quantitative variables were compared using the Mann–Whitney *U*-test. Categorical variables were compared between groups by the chi-square test and Fisher’s test. For the multiple comparisons, hypothesis testing was performed using the Bonferroni correction (the *p*-value divided by the total number of pairwise comparisons) to correct for the possibility that in the multiple comparisons, and the null hypothesis would be rejected by chance. All data were analyzed using Statistica v.19.0, and *p* < 0.05 was considered significant.

## Results

### Regional Distribution Characteristics of the WD Patients

Our patients came from most of the provinces and municipalities in China: Beijing (8 cases: 6 of neuropsychiatric form, 2 of hepatic form, 0 of Presymptomatic form), Tianjin (5 cases: 4,1,0), Shanghai (6 cases: 6,0,0), Chongqing (11 cases: 10,1,0), Anhui province (67,7,2), Fujian province (12,5,1), Gansu province (6,0,0), Guangdong province (4,1,2), Guangxi province (3,1,1), Guizhou province (3,0,0), Hebei province (15,3,0), Henan province (42,11,1), Heilongjiang province (10,3,0), Hubei province (24,2,0), Hunan province (16,1,0), Jilin province (14,3,1), Jiangsu province (46,8,2), Jiangxi province (23,3,0), Liaoning province (15,3,1), Inner Mongolia (4,0,1), Ningxia (2,1,0), Shandong province (45,3,0), Shanxi province (20,0,1), Shanxi province (13,1,1), Sichuan province (10,1,0), Xinjiang (3,0,0), Yunnan province (2,0,0), and Zhejiang province (27,8,0). The maximum number of patients was in Anhui Province (76 cases). For each province or city, the proportion of patients in neuropsychiatric form was the highest. thus, our data can reflect the situation of Chinese patients with hepatolenticular degeneration basically.

### Sex Differences in Clinical Parameters

Compared to female WD patients, male patients had a higher percentage in three forms (neuropsychiatric form: male/female = 263/189, hepatic form: male/female = 41/28, presymptomatic form: male/female = 10/4).

For all of the WD patients, the mean age of onset was 16.5 (range from 4 to 46 years), the mean age of diagnosis was 20.6 years (range from 6 to 61 years), and the mean disease latency was 4.3 years (range from 0.04 to 25 years). In neuropsychiatric form, women had the later age of both onset and being diagnosed as WD (*p* < 0.05), and the longer disease latency than men. In hepatic form, women had the later age of being diagnosed as WD and the longer disease latency than men, while women had the earlier age of onset than that of men. In presymptomatic form, women had the later age of both onset and diagnosis than men (**Table 1**).

**Table 1:** Clinical information of WD patients.

Clinical form	Male/female ratio	Age of onset	Age of diagnosis	Disease latency
Neuropsychiatric	263/189	All 16.8 (5–42)	All 21.0 (7–50)	All 4.5 (0.04–25)
(*n* = 452)		Male 16.6 (5–37)	Male 20.4 (7–43)	Male 4.1 (0.04–22)
		Female 17.1 (5–42)	Female 21.9 (9–50)	Female 5.0 (0.1–25)
Hepatic	41/28	All 15.6 (5–42)	All 18.7 (6–47)	All 3.2 (0.04–18)
(*n* = 69)		Male 15.7 (5–42)	Male 18.7 (6–47)	Male 3.1 (0.04–12)
		Female 15.5 (5–36)	Female 18.8 (8–37)	Female 3.3 (0.1–18)
Presymptomatic	10/4	All 12.6 (4–46)	All 16.1 (6–61)	All 3.4 (0.05–15)
(*n* = 14)		Male 13.9 (4–46)	Male 17.1 (6–61)	Male 3.1 (0.05–15)
		Female 9.5 (7–12)	Female 13.5 (7–21)	Female 4.0 (0.2–9)
All patients	314/221	All 16.5 (4–46)	All 20.6 (6–61)	All 4.3 (0.04–25)
(*n* = 535)		Male 16.4 (4–46)	Male 20.1 (6–61)	Male 3.9 (0.04–22)
		Female 16.7 (5–42)	Female 21.3 (7–50)	Female 4.7 (0.1–25)

*The disease latency: the time between age of onset and diagnosis.*

### MRI Findings

**FIGURE 1:**
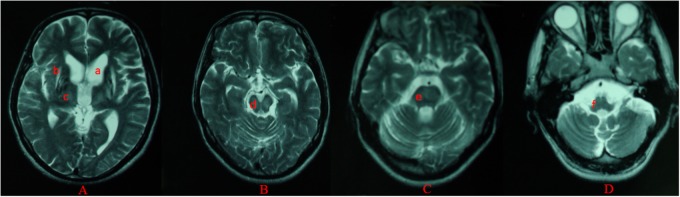
Different levels of brain MRI (T2WI) in a 33-year-old male **(A–D)**. axial images show ventricular widening **(a)** and hyperintensity in putamen **(b)**, thalamus **(c)**, mesocerebrum **(d)**, pons **(e)**, and medulla **(f)**.

**FIGURE 2:**
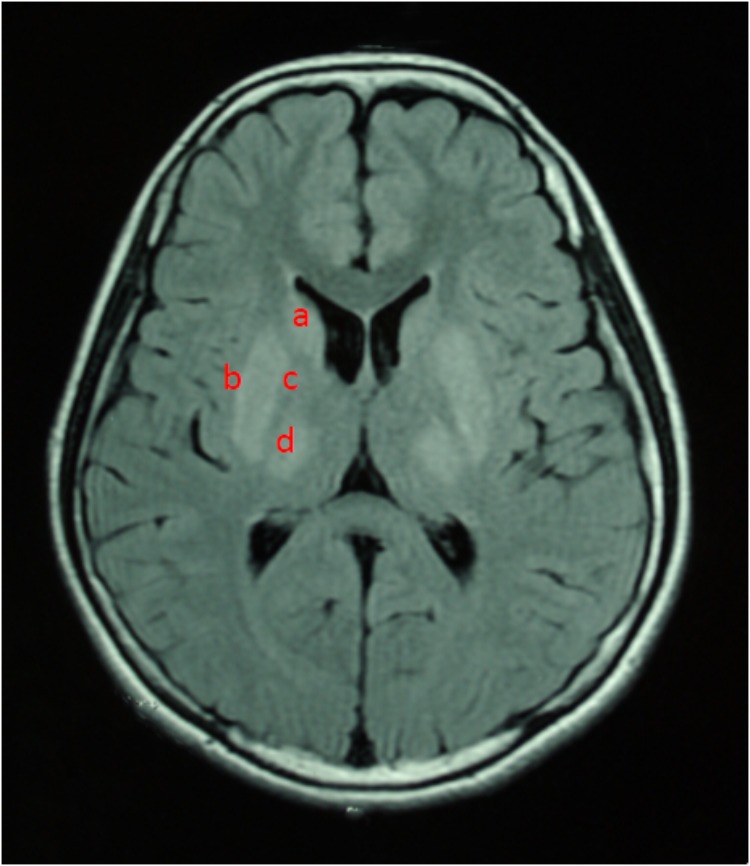
T2WI FLAIR image in a 12-year-old female. Axial images show hyperintensity in caudate nucleus **(a)**, putamen **(b)**, globus pallidus **(c)**, and thalamus **(d)**.

**FIGURE 3:**
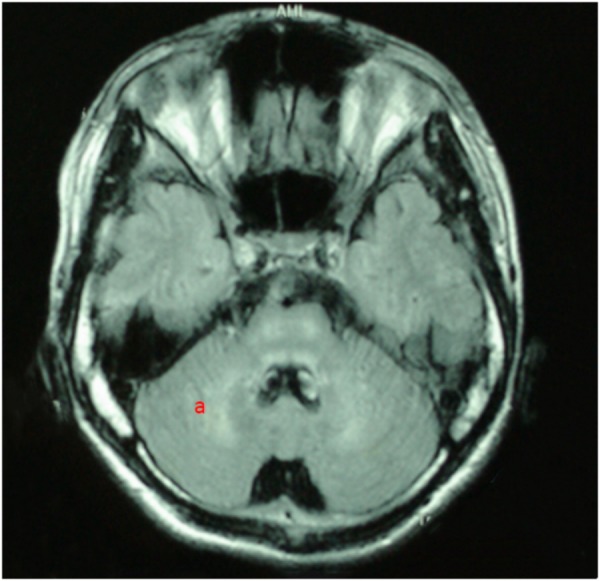
T2WI FLAIR image in a 19-year-old male. axial images show hyperintensity in cerebellum **(a)**.

For all of the patients, abnormal signal in the brain MRI was observed (**Figures 1**–**3**). We found lesions in the putamen (**Figures 1b**, **2b**) in 420 cases (men/women: 241/179), globus pallidus (**Figure 2c**) in 82 cases (49/33), caudate nucleus (**Figure 2a**) in 195 cases (106/89), thalamus (**Figures 1c**, **2d**) in 195 cases (110/85), mesocerebrum (**Figure 1d**) in 218 cases (130/88), pons (**Figure 1e**) in 139 cases (79/60), medulla (**Figure 1f**) in 3 cases (2/1), and cerebellum (**Figure 3a**) in 12 cases (6/6). In addition, we also found atrophy in the cerebellum or brain cortex in 56 cases (men/women = 28/28), and ventricular widening (**Figure 1a**) in 151 caess (87/64). Moreover, encephalomalacia was found in 16 cases (11/5) (**Tables 2**–**4**).

**Table 2:** Gender difference of the neuropsychitric WD patients in brain MRI.

Localization	Men (*n* = 263)	Women (*n* = 189)	*P*-value
Putamen (lesions)	198 (75%)	151 (80%)	0.249
Globus pallidus (lesions)	38 (14%)	28 (15%)	0.913
Caudate nucleus (lesions)	89 (34%)	76 (40%)	0.165
Thalamus (lesions)	92 (35%)	70 (37%)	0.653
Mesocerebrum (lesions)	105 (40%)	74 (39%)	0.869
Pons (lesions)	68 (26%)	50 (26%)	0.886
Medulla (lesions)	2 (1%)	1 (1%)	1.000
Cerebellum (lesions)	6 (2%)	6 (2%)	0.560
Cerebellum (atrophy)	24 (9%)	24 (13%)	0.224
Ventricular widening	72 (27%)	60 (32%)	0.314
Brain cortex (atrophy)	24 (9%)	24 (13%)	0.224
Encephalomalacia	10 (4%)	5 (3%)	0.498

*Date do not sum to 100% because of rounding errors; P for significance after Bonferroni correction: 0.004.*

Putamen was the most common site for lesions of three groups, while medulla lesions and cerebellum lesions were found only in patients with neuropsychitric form. Further, sex differences in ventricular widening percentage was found in hepatic patients (14/41 vs. 3/28; *p* < 0.05) (**Table 3**). However, there was no any other statistically significant gender difference in MRI data among the patients with neuropsychitric form and presymptomatic form.

**Table 3:** Gender difference of the hepatic WD patients in brain MRI.

Localization	Men (*n* = 41)	Women (*n* = 28)	*P*-value
Putamen (lesions)	34 (83%)	24 (86%)	1.000
Globus pallidus (lesions)	8 (20%)	4 (14%)	0.811
Caudate nucleus (lesions)	15 (37)	12 (43%)	0.600
Thalamus (lesions)	16 (39%)	13 (46%)	0.541
Mesocerebrum (lesions)	21 (51%)	12 (43%)	0.495
Pons (lesions)	9 (22%)	8 (29%)	0.531
Medulla (lesions)	0 (0%)	0 (0%)	–
Cerebellum (lesions)	0 (0%)	0 (0%)	–
Cerebellum (atrophy)	4 (10%)	3 (11%)	1.000
Ventricular widening	14 (34%)	3 (11%)	0.027
Brain cortex (atrophy)	4 (10%)	3 (11%)	1.000
Encephalomalacia	1 (2%)	0 (0%)	1.000

*Date do not sum to 100% because of rounding errors; P for significance after Bonferroni correction: 0.004.*

**Table 4:** Gender difference of the presymptomatic WD patients in brain MRI.

Localization	Men (*n* = 10)	Women (*n* = 4)	*P*-value
Putamen (lesions)	9 (90%)	4 (100%)	1.000
Globus pallidus (lesions)	3 (30%)	1 (25%)	1.000
Caudate nucleus (lesions)	2 (20%)	1 (25%)	1.000
Thalamus (lesions)	2 (20%)	2 (50%)	0.520
Mesocerebrum (lesions)	4 (40%)	2 (50%)	1.000
Pons (lesions)	2 (20%)	2 (50%)	0.520
Medulla (lesions)	0 (0%)	0 (0%)	–
Cerebellum (lesions)	0 (0%)	0 (0%)	–
Cerebellum (atrophy)	0 (0%)	1 (25%)	0.286
Ventricular widening	1 (10%)	1 (25%)	0.505
Brain cortex (atrophy)	0 (0%)	1 (25%)	0.286
Encephalomalacia	0 (0%)	0 (0%)	–

*Date do not sum to 100% because of rounding errors; P for significance after Bonferroni correction: 0.004.*

### Copper Metabolism

In neuropsychiatric form, the levels of serum ceruloplasmin and copper were higher in men than those in women. In hepatic form, the level of serum ceruloplasmin was higher in men than that in women, however, the level of serum copper in men was lower than that in women. In presymptomatic form, women had higher level of serum ceruloplasmin than men, while, the serum copper level was higher in men than that in women (**Table 5**).

**Table 5:** Serum ceruloplasmin, copper oxidase, and copper in WD patients.

Generally	Men (mean and SD)	Women (mean and SD)	*P*-value
**Neuropsychiatric form**			
Ceruloplasmin in μg/ml	72.28 ± 46.56	66.93 ± 36.58	0.172
Serum copper level in μmol/l	3.73 ± 2.54	3.54 ± 2.06	0.378
**Hepatic form**			
Ceruloplasmin in μg/ml	60.98 ± 26.11	55.85 ± 27.88	0.438
Serum copper level in μmol/l	3.48 ± 1.81	3.92 ± 2.03	0.344
**Presymptomatic form**			
Ceruloplasmin in μg/ml	57.59 ± 23.40	67.55 ± 41.01	0.570
Serum copper level in μmol/l	3.29 ± 1.83	3.20 ± 1.69	0.930

*P for significance after Bonferroni correction: 0.006.*

## Discussion

To our best knowledge, this study had the largest sample size of WD patients, focusing on sex difference in clinical parameters and brain MRI in Chinese WD patients. The main findings of our study included: (1) the neuropsychiatric form was the most common WD subtype (452 cases: 84.5%), and there were more male than female patients in each of three subtype groups; (2) Sex difference in ventricular widening was found only in hepatic form (*p* = 0.027).

The percentage of brain MRI abnormalities in our study (88.6%) is consistent with previous studies (73–88%) (Magalhaes et al., 1994; King et al., 1996; Saatci et al., 1997). We found that the neuropsychiatric form was the most subtype, and male patients had a higher percentage in three clinical forms. However, previous studies showed that the percentage of brain MRI abnormalities in female was higher than that in male, and the hepatic form was the most common subtype (Merle et al., 2007), which were different from our findings in this study. Differences in patient selection, diagnosis, and classification of clinical manifestations may lead to these discrepancies.

It was reported (Litwin et al., 2013) that more male than female patients showed cerebellar atrophy in neuropsychiatric form and cortical brain atrophy in presymptomatic form (*p* < 0.05), which speculated that the protective effect of estrogen and differences in gender-related clinical forms are potentially related to this differences. In this previous study, 204 newly diagnosed and untreated WD patients (neuropsychiatric form: 105 cases, hepatic form: 67 cases, presymptomatic form: 32 cases) was enrolled. In our study, sex difference was found in ventricular widening only in hepatic form favoring females (*p* < 0.05). The ventricular widening is also a sign of brain atrophy. We speculate that the estrogen may have the neuroprotective effect. A previous study showed that estrogen restrained the oxidative stress caused by reperfusion of ischemic tissue, and then reduced neuronal damage during middle cerebral artery occlusion (MCAO) (Shi et al., 2001). On the other hand, many studies showed that the inflammatory response in stroke, multiple sclerosis, Alzheimer’s disease, Parkinson’s disease, or amyotrophic lateral sclerosis play a critical role in the progress of neuronal injury. A previous study found that estrogen played the protective effect by influencing the production and bioactivity of inflammatory factors, such as TNFα, IL-1, and IL-6 (Czlonkowska et al., 2006). In an animal model of Parkinson’s disease, the neuroprotective effect of estrogen was observed in females, but not in males, suggesting that sex differences in nigrostriatal dopaminergic neurons react to injury may result from the differential actions of estrogen (Gillies et al., 2004). Taken together, we speculate that sex difference in ventricular widening in hepatic form may be related to neuroprotective effect of estrogen. However, we could not provide the reasonable explanation for sex difference in ventricular widening only in hepatic form, but not in other two forms, which deserves further investigation. Additionally, the iron metabolism in brain may be another important factor related to the sex difference in WD. For the age-related degenerative diseases, e.g., Alzheimer’s disease, Parkinson’s disease, and Dementia with Lewy Bodies, the brain ferritin iron may be a pathogenic factor (Rigamonti et al., 2005; Bartzokis et al., 2007). Clinical features may support this viewpoint, showing that women tend to display hepatic signs more often than men, while men tend to show neuropsychiatric symptoms more common than women (Litwin et al., 2012).

There were three limitations in our study. First, the number of the patients with hepatic form and presymptomatic form were much less compared with that of neuropsychiatric form, which may be attributed to the natural distribution of this disorder. Second, we did not test the urinary copper exertion level due to the lack of the 24 urinary copper exertion data, which will be remedied in future studies. Third, we did not measure the level of estrogen, and could not provide the reasonable explanation for sex difference in the ventricular widening in the hepatic form.

In summary, our findings demonstrated that the percentage of male patients was manifestly higher than that of female patients in three clinical forms of WD. The age of both onset and being diagnosed as WD were older in female than male patients, with the longer disease latency in females. The brain MRI showed lesions, atrophy, ventricular winding, and encephalomalacia in these WD patients, with marked lesions in putamen in both male and female patients. Sex difference was noted in the ventricular widening only in the hepatic form favoring female patients, which might be due to the protective effect of estrogen and the difference in iron metabolism. However, the pathological mechanisms underlying the sex differences in these findings are still unknown because of our retrospective design, which deserves further investigation by using the prospective and longitudinal design.

## Author Contributions

XhL and ZF participated in paper preparation. WT and XY participated in data processing. YQ and BL participated in data acquisition. XhL wrote the first draft. XsL participated in data acquisition and data processing. RY and YY designed the study.

## Conflict of Interest Statement

The authors declare that the research was conducted in the absence of any commercial or financial relationships that could be construed as a potential conflict of interest.
